# Multiplexed bacterial pathogen detection and clinical characteristics of orthopedic infection in hospitalized patients

**DOI:** 10.3389/fcimb.2024.1394352

**Published:** 2024-06-13

**Authors:** Yani Wang, Wenbo Xia, Ying Wang, Yanxiang Cui, Linhong Yu, Chao Liu, Dan Zhao, Xiaoxuan Guan, Yingdi Wang, Shanrui Wu, Jie Li, Yisong Li, Jianqiang Hu, Jie Liu

**Affiliations:** ^1^ School of Public Health, Qingdao University, Qingdao, China; ^2^ Department of Orthopedics, Qingdao Huangdao Traditional Chinese Medicine Hospital, Qingdao, China; ^3^ Department of Clinical Laboratory, Qingdao Huangdao Traditional Chinese Medicine Hospital, Qingdao, China; ^4^ Qingdao Medical College, Qingdao University, Qingdao, China

**Keywords:** orthopedic infection, bacterial pathogen, PCR, quantification, clinical manifestation, surgical site infection, *Staphylococcus aureus*

## Abstract

**Introduction:**

Accurate identification of the etiology of orthopedic infection is very important for correct and timely clinical management, but it has been poorly studied. In the current study we explored the association of multiple bacterial pathogens with orthopedic infection.

**Methods:**

Hospitalized orthopedic patients were enrolled in a rural hospital in Qingdao, China. Wound or exudate swab samples were collected and tested for twelve bacterial pathogens with both culture and multiplex real time PCR.

**Results and discussion:**

A total of 349 hospitalized orthopedic patients were enrolled including 193 cases presenting infection manifestations upon admission and 156 with no sign of infection. Orthopedic infection patients were mainly male (72.5%) with more lengthy hospital stay (median 15 days). At least one pathogen was detected in 42.5% (82/193) of patients with infection while 7.1% (11/156) in the patients without infection (*P* < 0.001). *S. aureus* was the most prevalent causative pathogen (15.5%). Quantity dependent pathogen association with infection was observed, particularly for *P. aeruginosa* and *K. pneumoniae*, possibly indicating subclinical infection. Most of the patients with detected pathogens had a previous history of orthopedic surgery (odds ratio 2.8, *P* = 0.038). Pathogen specific clinical manifestations were characterized. Multiplex qPCR, because of its high sensitivity, superior specificity, and powerful quantification could be utilized in combination with culture to guide antimicrobial therapy and track the progression of orthopedic infection during treatment.

## 1 Introduction

Orthopedic infection has been recognized as one of the major public health problems globally, which are usually complex and difficult to treat (Long et al., 2020; Premkumar et al., 2021; Metsemakers et al., 2023). It has a wide clinical spectrum, among which five types of bone and joint infection were usually categorized (Colston and Atkins, 2018; Kavanagh et al., 2018; Higgins et al., 2022), including septic arthritis, prosthetic joint infections, osteomyelitis, spinal infections, and diabetic foot osteomyelitis. Additionally, with the increased burden of traumatic injury (Chen et al., 2017), orthopedic infection associated with injury has drawn more and more attention, particularly fracture related infection (FRI) (Metsemakers et al., 2018; He et al., 2023).

Accurate diagnosis of orthopedic infection is critical for proper and timely patient managements, including antimicrobial treatment and surgery. The diagnostic schemes developed for orthopedic infection have been focused on both microbial etiology and host inflammatory response (Higgins et al., 2022). Culture followed by biochemical assays has been widely used as the gold standard. In combination with matrix-assisted laser desorption/ionization time-of-flight mass spectrometry (MALDI-TOF), identification of the cultured organisms has been greatly accelerated. MALDI-TOF alone was also utilized directly on clinical specimens independent of culture, particularly for the identification of microorganisms that are not culturable, however, the performance was found lower than that with culture enrichment in some cases (Kuo et al., 2020). With the rapid development of the molecular tools, polymerase chain reaction (PCR) based detection methods have been widely used, including conventional targeted PCR, broad range PCR followed by sequencing, and real time PCR or quantitative PCR (qPCR). These molecular methods have significantly shortened the turn-around-time and eliminated the false negative results by culture due to prior antibiotic treatment (Morgenstern et al., 2018).

Host responses are often assessed through detecting the biomarkers in serum or synovial fluid, histology, or radiology (Higgins et al., 2022). The inflammatory biomarkers, such as erythrocyte sedimentation rate (ESR) and C-reactive protein (CRP), can be readily included in the laboratory tests, but caution may have to be taken when interpreting the results due to the lack of specificity for infection. Histological analysis can provide confirmation for the inflammation, but can’t be utilized as a stand-alone method to rule out infection because of its low sensitivity. More advanced imaging examination such as ultrasound, radiology, computed tomography (CT) or magnetic resonance imaging (MRI) can facilitate the diagnosis, but such technologies are not always available (Jiang et al., 2015; Wang et al., 2017).

We have previously developed and validated a wide range of qPCR assays for molecular diagnostics of infectious diseases (Liu et al., 2014, 2016a, 2016b; Moore et al., 2019). Quantitative analysis approach facilitated the understanding of the etiology and epidemiology of these diseases (Liu et al., 2016c). The current study aimed to detect multiple bacterial pathogens by quantitative PCR (qPCR) directly on clinical samples of hospitalized orthopedic patients with or without suspected infection, so as to identify the main causative pathogens for such diseases and evaluate the clinical characteristics and risk factors of orthopedic infection.

## 2 Materials and methods

### 2.1 Participants

Inpatients with complete records of demographic, clinical and laboratory information, and with at least one specimen collected were enrolled in the Department of Orthopedics, Qingdao Huangdao Traditional Chinese Medicine Hospital from January 2021 to July 2023, excluding patients with bone tuberculosis and diabetic foot. Their infection statuses were evaluated by the clinicians upon admission, which was used to divide the participants into two groups, one with clinically identified infection manifestation and the other with no sign of infection. A patient was categorized into Group A (suspected infection) with any one or more of the 9 scenarios below (**Table 1**).

- Open injury with severely contaminated wound;
- Hospital admission was more than 24 hours after the onset of open injury;
- Skin redness, swelling, high skin temperature, pain with dermal ulcer, fistula, sinus track, or purulent exudate;
- Joint redness, swelling, and burning pain accompanied by elevated body temperature or increased white blood cells count (WBC), neutrophils, CRP, ESR, or other inflammatory biomarkers;
- Abnormal joint puncture fluid or purulent joint fluid;
- Joint or limb abscess with visible pus;
- Post orthopedic surgery, the incision was red, swollen, burning, painful, and ruptured with non-bloody exudate;
- Blood culture was recommended when the body temperature continued to rise with no other infection and hematogenous dissemination was considered.
- Clinical samples were collected during the operation of confirmed infection cases.

**Table 1:** Demographic characteristics and clinical diagnosis of patients enrolled in the current study.

	Total (n = 349)	Group A (n=193)	Group B (n=156)	*P*-value
Gender				0.288
Male	245 (70.2%)	140 (72.5%)	105 (67.3%)	
Female	104 (29.8%)	53 (27.5%)	51 (32.7%)	
**Age (years)**	50.6 ± 16.3	51.6 ± 16.6	49.4 ± 15.8	0.210
**Duration of hospitalization (days)**	14.0 (8.0, 23.0)	15.0 (8.0, 30.5)	13.0 (8.0, 18.8)	**0.035**
Clinical diagnosis				<0.001
Fracture caused by trauma	144 (41.3%)	56 (29.0%)	88 (56.4%)	
Open injury	47 (13.5%)	31 (16.1%)	16 (10.3%)	
Closed injury	17 (4.9%)	6 (3.1%)	11 (7.1%)	
Post operation	42 (12.0%)	29 (15.0%)	13 (8.3%)	
Suspected infection	61 (17.5%)	57 (29.5%)	4 (2.6%)	
Bone diseases	26 (7.4%)	7 (3.6%)	19 (12.2%)	
Other orthopedic diseases	12 (3.4%)	7 (3.6%)	5 (3.2%)	
Injury sites				0.585
Upper limbs	67 (19.2%)	40 (20.7%)	27 (17.3%)	
Lower limbs	195 (55.9%)	108 (56.0%)	87 (55.8%)	
Trunk	18 (5.2%)	11 (5.7%)	7 (4.5%)	
Multiple parts of the body	68 (19.5%)	33 (17.1%)	35 (22.4%)	
Head and face	1 (0.3%)	1 (0.5%)	0	
Clinical symptoms				
Redness	75 (21.5%)	67 (34.7%)	8 (5.1%)	**<0.001**
Purulent exudate	23 (6.6%)	23 (12.0%)	0	**<0.001**
Diabrosis	50 (14.3%)	47 (24.4%)	3 (1.9%)	**<0.001**
Seepage	72 (20.6%)	67 (34.7%)	5 (3.2%)	**<0.001**
High skin temperature	56 (16.0%)	49 (25.4%)	7 (4.5%)	**<0.001**
Open wound	105 (30.1%)	72 (37.3%)	33 (21.2%)	**0.002**
Necrosis	10 (2.9%)	10 (5.2%)	0	**0.010**
Restricted activity	262 (75.1%)	129 (66.8%)	133 (85.3%)	**<0.001**
Subcutaneous hemorrhage	42 (12.0%)	15 (7.8%)	27 (17.3%)	**0.006**
Malformed	65 (18.6%)	26 (13.5%)	39 (25.0%)	**0.006**
Fever	3 (0.9%)	1 (0.5%)	2 (1.3%)	0.853
Swelling	228 (65.3%)	122 (63.2%)	106 (67.9%)	0.355
Bleeding	104 (29.8%)	57 (29.5%)	47 (30.1%)	0.904
Pain	320 (91.7%)	175 (90.7%)	145 (92.9%)	0.444
Tissues exposed	22 (6.3%)	14 (7.3%)	8 (5.1%)	0.417
Contaminated wound	52 (14.9%)	30 (15.5%)	22(14.1%)	0.707
Bone exposure	12 (3.4%)	5 (2.6%)	7 (4.5%)	0.334
Effusion	15 (4.3%)	10 (5.2%)	5 (3.2%)	0.365
Implant exposure	1 (0.3%)	1 (0.5%)	0	1
Unhealed incision	8 (2.3%)	7 (3.6%)	1 (0.6%)	0.135
Poor circulation	30 (8.6%)	18 (9.3%)	12 (7.7%)	0.588

Comparison was performed between group A and B.Statistical significance was marked in bold.

The patients with the following scenarios were categorized into Group B.

- Sampling the open wound after debridement to rule out infection;
- Routine culture when pulling out drainage tube post orthopedic surgery;
- Routine culture of bloody exudate post orthopedic surgery;
- Bacterial culture was performed on turbid joint puncture fluid to rule out infection;
- Samples of joint or limb hematoma (no obvious pus) to rule out infection.

### 2.2 Specimen collection

Two swab samples (dry or in transport medium) or exudate samples were simultaneously collected from all the patients upon enrollment following the standard hygiene procedure. One sample was subjected to bacterial culture immediately, and the other stored at −80°C until qPCR testing.

### 2.3 Bacterial culture

Bacterial culture was performed with the swab samples collected in transport medium by plating blood agar or China blue agar plates (babio, Jixian, China), and incubation at 37°C for 24 hours. Single colonies were picked and tested with VITEK^®^ 2 COMPACT automatic microbiology analyzer for bacterial identification (bioMérieux, Marcy L’Etoile, France).

### 2.4 Nucleic acid extraction

Swab samples were resuspended in 200μl of PBS buffer (pH 7.2). Nucleic acid was extracted using manual bacterial genomic DNA extraction Kit (centrifugal column type, BioTeke, China) or automated IndiSpin^®^ QIAcube^®^ HT Pathogen Kit (Leipzig, Germany). Each sample was spiked with an external control, phocine herpesvirus (PhHV) to monitor the extraction and amplification efficiency. One extraction blank was included per batch of extraction to rule out laboratory contamination.

### 2.5 PCR tests

Real-time PCR primers and probes were adapted from publications (Kodani et al., 2011; Diaz et al., 2013) or in house developed and validated, as listed in **Table 2**. Four 3-plex real-time PCR panels were formulated for the detection of 12 pathogens previously reported in orthopedic infection (Liebling et al., 1994; Wang et al., 2018; Xie et al., 2020; Bruun et al., 2021; Kawaguchi et al., 2022), including *Staphylococcus aureus*, *Acinetobacter baumannii*, *Pseudomonas aeruginosa*, *Escherichia coli*, *Haemophilus influenzae*, *Neisseria meningitidis*, *Klebsiella pneumonia*, *Klebsiella aerogenes*, *Enterobacter cloacae, Streptococcus pneumoniae, Streptococcus pyogenes*, and *Streptococcus agalactiae*. The panels were tested for linearity, lower limit of detection (LOD), repeatability, reproducibility, and analytical specificity as described in **Supplementary Table S1**. Each 10-μl multiplex PCR contained 5 μl of 2×AgPath-ID™ One-step reverse transcription PCR (RT-PCR) buffer (Life Technologies, USA), 0.4 μl of enzyme mix, primers and probes at the concentrations indicated in **Table 2**, and 2 μl of nucleic acid extract. Cycling conditions included 20 min of reverse transcription at 45°C, 10 min of initial denaturation at 95°C, and 45 cycles of 15 s at 95°C and 1 min at 60°C. One pooled positive control and a no template negative control were included in each run. qPCR panels were performed and analyzed with QuantStudio 7 Flex real-time PCR system with software v1.7 (Thermofisher, USA). Quantification cycle (Cq) 35 was used as the cutoff for positivity unless otherwise stated.

**Table 2:** Primer and probe sequences of multiplex qPCR.

	Pathogen	Target gene	Sequence (5’-3’)	Amplicon length (bp)	Final concentration (nM)
**Panel 1**	*S. aureus*	*sodA*	F-GCGTGTTCCCATACGTCTAAACC	79	900
			R-TTGTGACTACACCAAACCAAGATAAT		900
			P-FAM-CATTAACTGAGGGTAAAACACC		250
	*P. aeruginosa*	*gyrB*	F-GTCTCGGTGGTGAACG	182	540
			R-TGGATGTTGCTGAAGGTCTC		540
			P-VIC-TCCGTCGCCACAACAAGGTCTGGGAA		150
	*A. baumannii*	*bla*_OXA_	F-TATTTTTATTTCAGCCTGCTCACCTT	271	900
			R-AAATACTTCTGTGGTGGTTGCCTTA		900
			P-NED-TGACTGCTAATCCAAATCACAGCGCTTCA		250
**Panel 2**	*E. coli*	*uidA*	F-GAGCATCAGGGTGGCTATACG	113	900
			R-ATAGTCTGCCAGTTCAGTTC		900
			P-FAM-TACGGCGTGACATCGGCTTCAAATG		250
	*N. meningitidis*	*sodC*	F-CTGTGAGCCAAAAGAAAAAGAA	164	900
			R-GATTTGTTGCTGTGCCATCAT		900
			P-VIC-CGCAGGCGGTCACTGGGATC		250
	*H. influenzae*	*hpd*	F-TGCGGTAGTGTTAGAAAATGGTATTAT	116	900
			R-GGACAAACATCACAAGCGGTTA		900
			P-NED-TATCAATACTACAACGAGACGCAA		250
**Panel 3**	*K. pneumonia*	Diguanylate cyclase encoding gene	F-TGCAGATAATTCACGCCCAG	133	900
			R-ACCCGCTGGACGCCAT		900
			P-FAM-CCACCACGCTCATCTGTTTCGCC		250
	*E. cloacae*	ECL1133*	F-AAAACCTTATCCGCGACGTT	89	900
			R-CGGCTTTTCCTGAAATGAGA		900
			P-VIC-CAGGCATATTCATGCCAACC		250
	*K. aerogenes*	hdc*	F-ATCAGGACCTCAGGCGAAC	85	900
			R-TCTCAGCGATATTATCAACAGC		900
			P-NED-ATTTTCGCCAATATWGGCAC		250
**Panel 4**	*S. pneumoniae*	*lytA*	F-ACGCAATCTAGCAGATGAAGCA	75	900
			R-TCGTGCGTTTTAATTCCAGCT		900
			P-FAM-CCGAAAACGCTTGATACAG		250
	*S. pyogenes*	spy1258	F-GCACTCGCTACTATTTCTTACCTCAA	98	900
			R-GTCACAATGTCTTGGAAACCAGTAAT		900
			P-VIC-CCGCAACTCATCAAGGATTTCTGTTACCA		250
	*S. agalactiae*	*cfb*	F-GGGAACAGATTATGAAAAACCG	126	900
			R-AAGGCTTCTACACGACTACCAA		900
			P-TAMRA-AGACTTCATTGCGTGCCAACCCTGAGAC		250

* These assays were developed and validated in the current study. F, forward primer; R, reverse primer; P. TaqMan probe.

### 2.6 Ethics statement

The study was reviewed and approved by the Ethics Committee of Qingdao University and Qingdao Huangdao Traditional Chinese Medicine Hospital. The patients provided written informed consent. All procedures were performed in accordance with the 1964 declaration of Helsinki and later amendments.

### 2.7 Statistical analysis

The measured data that conformed to a normal distribution were expressed in terms of the mean (*X*), and compared using a *t-*test. Measurements that fell within a skewed distribution were expressed as medians (interquartile range, IQR), and compared using a Mann-Whitney *U* test. A categorical variable was expressed in numbers or percentages, and proportions were compared using either Pearson’s chi-square or Fisher’s exact test. Receiver operating characteristic (ROC) analysis was used to evaluate the performance of qPCR against bacterial culture and determine the association of pathogen detection with infection status. Two-tailed *P* values were calculated, and values of < 0.05 were considered statistically significant. All analyses were performed using IBM SPSS Statistics version 26.

## 3 Results

### 3.1 Clinical features of patients with suspected infection

A total of 349 inpatients were enrolled by Department of Orthopedics, and according to the clinical manifestations assessed by the clinicians upon admission, infection was clinically confirmed or suspected requiring laboratory diagnosis in 193 patients (group A) (**Table 1**). The rest 156 patients were classified into group B without any suspected infection, for which routine screening was performed to rule out infection. More male patients (245, 70.2%) were enrolled than females (104, 29.8%), with a male to female ratio of 2.64 in group A and 2.06 in group B (*P* = 0.288) (**Table 1**). The median age was 52 years in group A and 49 years in group B (*P* = 0.210). The length of hospital stay was statistically different, with a median of 15 (IQR 8.0, 30.5) days in group A and 13 (IQR 8.0, 18.8) in group B (*P* = 0.035). Fracture accounted for the most common diagnosis on admission (144, 41.3%), followed by suspected infection (61, 17.5%), open injury (47, 13.5%), post operation (42, 12.0%), bone diseases (26, 7.4%), closed injury (17, 4.9%), and other orthopedic diseases (12, 3.4%). Among the injured sites, the lower limbs were the most common, accounting for 55.9%. No difference in the injured sites was observed between the two groups. **Table 1** showed the number of patients exhibited the clinical symptoms defining the two groups. The most common symptoms included open wound (37.3%), redness (34.7%), seepage (34.7%), high skin temperature (25.4%), diabrosis (24.4%), purulent exudate (12.0%) and necrosis (5.2%) in the infection group. Other symptoms had similar distribution in the two groups, such as fever, swelling, bleeding, pain, tissues exposed due to wound opening, etc. Majority of the patients in group B tended to have restricted activity (85.3% versus 66.8% in group A, *P* < 0.001). The laboratory tests (**Supplementary Table S2**) from a number of the patients showed elevated biomarkers for bacterial infections, such as white blood cell count (WBC, 18.4%), absolute neutrophil count (ANC, 25.4%), procalcitonin (PCT, 61.3%), ESR (68.0%), CRP (61.3%), and D-dimer (34.0%). There was no statistical difference between the two groups except high PCT in more patients of group B (*P* = 0.030).

### 3.2 Pathogen detection by bacterial culture

As shown in **Table 3**, 61 (31.6%) patients in group A were positive by culture for one of the 12 pathogens interrogated, with *S. aureus* predominantly detected at 20.7% (40/193), followed by *E. coleacae* complex (ECC) at 3.1% (6/193), and *A. baumannii* complex (ABC)*, E. coli, P. aeruginosa* at 2.6% (5/193). Three (1.9%, *P* < 0.001) patients in group B were found positive, including 2 cases of *S. aureus* and one case of *E. cloacae* complex. Six of the ECC strains were identified as *E. hormaechei*, and one as *E. roggenkampii* through whole genome sequencing (data not shown). Similarly, three ABC strains were further identified as *A. baumannii*, two as *A. pittii*, and one as *A. soli*.

**Table 3:** Comparison of the bacterial pathogen detection between culture and qPCR in the two groups.

Pathogen	Total (349)	Group A (193)	Group B (156)	*P*-value	OR (95% CI)
*S. aureus* Total	54 (15.5%)	50	4	**<0.001**	13.3 (4.7, 37.7)
culture+	42	40	2	**<0.001**	20.1 (4.8, 84.8)
qPCR+	47	43	4	**<0.001**	10.9 (3.8, 31.1)
qPCR+ Cq	30.5 (26.4, 32.6)	30.9 (26.9, 32.7)	27.4 (25.1, 31.0)	0.253	–
*A. baumannii* Total	7 (2.0%)	7	0	**0.043**	1.9 (1.7, 2.0)
culture+	5	5	0	**0.043**	-
qPCR+	4	4	0	0.071	-
qPCR+ Cq	31.1 ± 3.0	31.1 ± 3.0	-	-	-
*P. aeruginosa* Total	9 (2.6%)	6	3	0.487	1.6 (0.4, 6.7)
culture+	5	5	0	**0.043**	–
qPCR+	8	5	3	0.956	–
qPCR+ Cq	27.2 ± 6.8	23.8 ± 6.0	33.0 ± 3.3	**<0.001**	–
*E. coli* Total	15 (4.3%)	14	1	**0.002**	12.1 (1.6, 93.2)
culture+	5	5	0	**0.043**	-
qPCR+	14	13	1	**0.004**	11.2 (1.4, 86.5)
qPCR+ Cq	30.1 ± 4.0	29.9 ± 4.0	-	-	-
*N. meningitidis* Total	1 (0.3%)	1	0	1	–
culture+	–	0	0	–	–
qPCR+	1	1	0	1	–
qPCR+ Cq	–	–	–	–	–
*K. pneumoniae* Total	17 (4.9%)	13	4	0.072	2.7 (0.9,8.6)
culture+	3	3	0	0.256	-
qPCR+	15	11	4	0.151	-
qPCR+ Cq	31.6 ± 3.4	30.5 ± 3.4	34.6 ± 0.1	**0.003**	-
*K. aerogenes* Total	3 (0.9%)	3	0	0.327	–
culture+	1	1	0	1	–
qPCR+	3	3	0	0.327	–
qPCR+ Cq	32.9 ± 2.4	32.9 ± 2.4	–	–	–
*E. cloacae* Total	10 (2.9%)	9	1	**0.025**	7.6(1.0, 60.5)
culture+	7	6	1	0.136	-
qPCR+	3	3	0	0.327	-
qPCR+ Cq	33.0 ± 2.3	33.0 ± 2.3	-	-	-
*S. pyogenes* Total	1 (0.3%)	1	0	1	–
culture+	–	0	0	–	–
qPCR+	1	1	0	1	–
qPCR+ Cq	–	–	–	–	–
*S. agalactiae* Total	3 (0.9%)	3	0	0.327	-
culture+	1	1	0	1	-
qPCR+	3	3	0	0.327	-
qPCR+ Cq	29.3 ± 7.3	29.3 ± 7.3	-	-	-
Sum Total	93 (26.6%)	82	11	**<0.001**	9.7 (5.0,19.1)
culture+	64	61	3	**<0.001**	23.6 (7.2, 76.9)
qPCR+	82	71	11	**<0.001**	7.7 (3.9, 15.1)
qPCR+ Cq	30.5 (27.1, 32.8)	30.2 (27.0, 32.5)	33.9 (28.9, 34.9)	**0.048**	–

-, not applicable. The detection (culture+, qPCR+, and Total of culture and qPCR combined) and qPCR Cqs were shown. Pathogen Cqs that conformed to a normal distribution were expressed as mean with standard deviation, while those fell within a skewed distribution were expressed as medians (IQR).Statistical significance was marked in bold.

### 3.3 Pathogen detection by multiplex real time qPCR panels

Multiplex qPCR panels underwent standard optimization and validation (**Supplementary Table S1**). Depending on the target, the qPCR efficiency ranged from 80.1% to 100.0%. The LOD was 10–20 copies per qPCR reaction, which corresponded to an averaged Cq of 38.5. These assays demonstrated excellent repeatability and reproducibility, and an overall 97.1% analytical sensitivity and 100% analytical specificity on culture isolates.

With Cq cutoff of 38.5 determined at LOD, qPCR detected all culture positives except one case of *S. aureus*, *P. aeruginosa*, *K. pneumoniae*, and *E. coli* each. The *E. cloacae* assay, specifically targeting *E. cloacae* only, didn’t detect 7 non-*E. colacae* ECC culture positives. *A. baumannii* assay, specifically targeting *A. baumannii* only, detected two of ABC positive by culture, which were identified as *A. baumannii*, but not those identified as *A. pittii* and *A. soli*. These corresponded to an overall clinical sensitivity of 93.2% (55/59). However, qPCR yielded 132 excessive pathogen detections compared to culture. PCR followed by amplicon sequencing was then performed to confirm these results. Using the sequencing results as reference, ROC analysis showed that the results with Cq < 35 were reliably confirmed (data not shown). Cq cutoff of 35, which yielded a moderate consistency between qPCR and culture (Kappa = 0.712, *P* < 0.001), was used for further analysis. As shown in **Table 3**, 36.8% (71/193) of the patients in group A were tested positive for any of the 12 pathogens, with 15 (7.8%) for more than one pathogen. *S. aureus* was present in 40% of the mixed infections. Although there was no apparent infection, 11 (7.1%, *P* < 0.001) patients in group B were positive for *S. aureus* (4), *P. aeruginosa* (3), *E. coli* (1), and *K. pneumoniae* (4). The overall Cq values of group A (median 30.2, IQR (27.0, 32.5)) were significantly lower than those of group B (33.9, IQR (28.9, 34.9), *P* = 0.048), particularly for *P. aeruginosa* and *K. pneumoniae*. ROC analysis showed that Cq 28.7 and 34.4 served better separation of infection and non-infection groups for *P. aeruginosa* and *K. pneumoniae*, respectively. Consistent with the culture results, *S. aureus* was also the most prevalent pathogens by PCR (43, 22.3%). No positive was detected for *H. influenzae* and *S. pneumoniae* by either culture or qPCR. Combining the culture and PCR results, the detection of *S. aureus*, *E. coli*, *E. cloacae*, *A. baumannii*, *K. pneumonia*, and *P. aeruginosa* was significantly different between the two groups (**Table 3**).

### 3.4 Clinical presentations related to bacterial pathogens

Comparing the clinical information of the patients with detection of any of the 12 pathogens by either culture or PCR (n = 81) with those without any of the pathogens detected (n = 95), statistically significant difference was observed in gender, clinical manifestations such as seepage and higher skin temperature, and surgery history (**Supplementary Table S3**). Bleeding, poor circulation, and tissues exposed seemed to be more common in the patients without any pathogen detected. Men are more susceptible to these causative agents, more than twice as likely to be infected as women (*P* = 0.027). Interestingly, sialic acid (SA) was significantly increased in the patients with pathogens identified (OR 2.4, *P* = 0.006), dominant by *S. aureus* (65%, 26/40; OR 3.1, *P* < 0.001). As the most prevalent pathogen (n = 54), *S. aureus* showed association with redness (odds ratio, OR 5.2, *P* < 0.001), purulent exudate (OR 8.7, *P* < 0.001), diabrosis (OR 3.7, *P* < 0.001), seepage (OR 3.8, *P* < 0.001), high skin temperature (OR 5.3, *P <* 0.001) and unhealed incision (OR 6.4, *P* = 0.027). *E. coli* (n = 15) likely caused diabrosis (OR 3.5, *P* = 0.034) and seepage (OR 5.6, *P* = 0.002). *K. pneumoniae* (n = 13) showed unique association with necrosis (OR 14.6, *P* = 0.022) while *E. cloacae* strongly associated with purulent exudate (OR 14.5, *P* = 0.005) (**Table 4**).

**Table 4:** The association of bacterial pathogens with clinical symptoms.

Clinical symptom	*S. aureus*	*E. coli*	*K. pneumoniae*	*E. colacae*
Redness	**5.2 (2.7, 9.8)**	2.0 (0.6, 6.7)	1.7 (0.4, 6.4)	**3.7 (1.0, 13.9)**
Purulent exudate	**8.7 (3.2, 23.6)**	2.4 (0.3, 21.0)	**6.2 (1.1, 33.2)**	**14.5 (3.1, 68.2)**
Diabrosis	**3.7 (1.8, 7.7)**	**3.5 (1.0, 11.9)**	**4.3 (1.2, 15.0)**	1.1 (0.1, 8.8)
Seepage	**3.8 (1.9, 7.3)**	**5.6 (1.9, 16.5)**	**4.0 (1.2, 13.0)**	1.6 (0.3, 7.9)
High skin temperature	**5.3 (2.7, 10.6)**	0.6 (0.1, 4.7)	**5.2 (1.6, 17.2)**	3.6 (0.9, 14.8)
Fever	–	–	10.1 (0.9, 119.1)	–
Tissues exposed	0.2 (0.0, 1.8)	–	–	–
Contaminated the wound	0.1 (0.0, 0.8)	0.4 (0.0, 3.0)	1.0 (0.2, 4.6)	3.6 (1.0, 13.4)
Open wound	0.6 (0.3, 1.3)	0.9 (0.3, 2.9)	1.1 (0.3, 3.6)	**5.7 (1.4, 22.6)**
Necrosis	3.1 (0.5, 19.0)	–	**14.6 (2.2, 96.5)**	–
Swelling	1.1 (0.6, 2.0)	1.3 (0.4, 4.3)	1.1 (0.3, 3.7)	0.7 (0.2, 2.7)
Bleeding	0.2 (0.1, 0.6)	0.5 (0.1, 1.9)	0.9 (0.3, 3.1)	3.1 (0.9, 11.4)
Pain	0.6 (0.2, 1.6)	0.5 (0.1, 2.3)	0.9 (0.1, 7.3)	–
Malformed	0.2 (0.1, 0.8)	1.4 (0.4, 4.6)	1.2 (0.3, 4.4)	1.0 (0.2, 4.7)
Restricted in activity	0.4 (0.2, 0.7)	0.5 (0.2, 1.6)	0.3 (0.1, 1.0)	0.4 (0.1, 1.5)
Bone exposure	–	–	–	2.4 (0.3, 20.3)
Subcutaneous hemorrhage	0.6 (0.2, 1.6)	0.9 (0.2, 4.2)	–	–
Effusion	0.9 (0.2, 4.2)	–	2.0 (0.2, 16.5)	–
Implant exposure	–	–	–	–
Unhealed incision	**6.4 (1.4, 29.6)**	–	–	8.9 (0.8, 94.4)
Poor circulation	0.4 (0.1, 1.6)	–	–	1.1 (0.1, 8.8)

The odds ratios with 95% confidence interval were presented. The analysis was limited to the pathogens with the number of positive detection ≥ 10.Statistical significance was marked in bold.

## 4 Discussion

In this study, we enrolled 349 hospitalized patients from Orthopedics Department in a rural hospital with its patient population being mostly of relatively low socioeconomic status. Their initial clinical diagnosis upon admission were diverse, but with majority related to traumatic injury or operation (72.7%, 250/349). With further evaluation based on their clinical presentations, 55.3% (193/349) of these patients were diagnosed as suspected infection. Twelve bacterial pathogens were tested by both culture and qPCR on the wound samples, including commonly reported *S. aureus*, *E. coli*, *K. pneumoniae*, *P. aeruginosa*, *A. baumannii*, and *E. cloacae*, and less common bacteria such as *H. influenzae*, *N. meningitidis, K. aerogenes*, *S. pneumoniae, S. pyogenes*, and *S. agalactiae*. The two methods exhibited good correlation. With a conserved qPCR Cq cutoff at 35, where the positive results could be reproducibly confirmed with amplicon sequencing, pathogens were detected in 36.8% of patients with suspected infection. *S. aureus* accounted for 22.3% as the main cause, followed by other common pathogens, which was consistent with the findings on fracture associated infection in various regions in China (Wang et al., 2021a). Additionally, four of the less common pathogens, i.e. *S. agalactiae, N. meningitidis, K. aerogenes*, and *S. pyogenes* were detected. Further surveillance on these bacteria and beyond may be important to evaluate their clinical significance.

Worth clarifying is that the qPCR assay used for the detection of *E. cloacae* was species specific while bacterial culture identifies *Enterobacter cloacae* complex without further discrimination, which composes of *E. cloacae, E. asburiae, E. kobei, E. hormaechei, E. roggenkampii, E. ludwigii*, and *E. bugandensis.* Whole genome sequencing results showed that ECC isolates identified by culture in the current study were all non *E. cloacae* species, i.e. *E. hormaechei* and *E. roggenkampii*, indicating the needs of assays inclusive for ECC or specific for the major species with clinical relevance in the future surveillance. There may be a similar requirement for *Acinetobacter baumannii* complex, but likely to a less extent, as *A. baumannii* was found to be the predominant species.

While majority of the detection took place in group A, pathogens were identified, mostly by qPCR due to its high sensitivity, in 11 cases without apparent infection (group B). There was significant difference in pathogen quantities between group A and B. Furthermore, through ROC analysis, cutoffs of Cq 28.7 and Cq 34.4 perfectly separated the distribution of *P. aeruginosa* and *K. pneumoniae* in the two groups, respectively. This might indicate that pathogen quantity played an important role in infection severity and disease progression. The elucidation of the importance of such “subclinical infection” may require further investigation. Worth noting is that a big portion of samples (n = 132) yielded Cq values between 35 and 38.5 for at least one of the 12 pathogens. As the previous study ruled out the possibility of “false positive” resultant from dead bacteria in the infected wound (Kaplan et al., 2018), another possible source of such low level detections could be skin contamination during sample collection although standard hygiene procedure was applied. qPCR has the advantage of modulating the detection sensitivity and stringency for different purposes, most importantly clinical relevance, such as in childhood diarrhea and pneumonia (Liu et al., 2016c; Pneumonia Etiology Research for Child Health (PERCH) Study Group, 2019). Further longitudinal sampling combined with qPCR quantification might help resolve whether these results could reveal any meaningful implication on subclinical progression.

Gender was a risk factor for bacterial pathogens detection, with OR of 2.2 for male. Most patients with detected pathogens had a previous history of orthopedic surgery (OR 2.8, *P* = 0.038). Almost any of the bacteria that colonize at the anatomic sites can become an opportunistic pathogen causing infection (Garcia et al., 2019). The distribution of normal skin microbiota varies by individual. In general, surgical site infection (SSI) is greatly underreported, especially post discharge. For example, the rates of SSI in arthroplasties have been reported between 1% and 5%. However, post-discharge follow-up revealed an increase of 450% for SSI (Carneiro et al., 2014). In the current study, 11 of 13 cases with pathogen detected in the patients with no symptomatic infection (group B) were associated with surgery. This strongly suggested the necessity of pathogen screening post-surgery even without any apparent infection sign. Repeatedly, highly sensitive but modulable detection would be preferred.

The main clinical manifestations of patients in the pathogen positive group were exudation and high skin temperature while the top pathogens demonstrated distinct association with the clinical symptoms. The criteria used by the clinicians in the current study seemed to perform well to differentiate infection purely based on clinical manifestations, although they were admittedly subjective. In the patients with suspected infection, several biomarkers (**Supplementary Table S2**) that were previously reported to be relevant with orthopedic infection showed significant difference from those without apparent infection, such as increased absolute value of immature granulocytes (Wu et al., 2019), platelet count (Liu et al., 2022a), alanine aminotransferase (Talsnes et al., 2012; Huang et al., 2023), globulin (α1 and α2) (Ye et al., 2020), γ-glutamyl transpeptidase (Fisher et al., 2015), total cholesterol (Li et al., 2023), sialic acid (SA) (Cui et al., 2014), and decreased albumin (Fisher et al., 2015), Ratio of albumin to globulin (Wang et al., 2021b), prealbumin (Liu et al., 2022b), high-density lipoprotein cholesterol (Zhang et al., 2021). However, in the current study, none of the laboratory tests was found to be associated with infection, such as CRP, ESR, and PCT. The lack of specificity of these biomarkers has been recognized as the major obstacle for wide practical use. Interestingly, patients in the pathogen positive group had higher sialic acid (*P* < 0.05), and the OR value suggested that the risk of high sialic acid in patients infected with pathogens was 2.4 times higher than that in no pathogen detection group. Sialic acid has recently been found to play an important role in *S. aureus* airway infection by triggering virulence reprogramming (Ding et al., 2023). Whether these could be use as the guidance or biomarker for clinical diagnosis would require extensive verification.

The limitations of this study included, firstly, the study focused on hospitalized patients with orthopedic infections from a single center, which might introduce bias if the results were different from those of the outpatients and of other facilities. However, this study proved that clinical symptom based grouping criteria were mostly accurate for infection determination, and quantification brought more power to the analysis, although external validation of the quantitative findings is needed. Secondly, the sample size of this study was relatively small, particularly for the less common pathogens, which requires larger population for more comprehensive evaluation. Increased sample size from the continuation of such surveillance would also enable discrimination of etiology for orthopedic infection of specific mechanisms, such as fracture related infection, periprosthetic joint infection, osteomyelitis, etc. Meanwhile, continuous surveillance will allow us to track the temporal variation and evaluate the potential impact of SARS-CoV-2 pandemic during the research period of this study. Lastly, only twelve bacterial pathogens were analyzed in the current study, and about 30% of the samples from the patients with suspected infection were negative for all the pathogens even with Cq cutoff at 40.

Multiplex qPCR enabled faster, more sensitive and specific detection and quantification of the pathogens in orthopedic infection. Quantitative analysis improved the discrimination of symptomatic infection, potentially subclinical infection without any symptom, and no infection. This method could be utilized in combination with culture in such settings to guide antimicrobial therapy. It might be particularly useful for tracking the progression of infection during treatment. More extensive screening for various microorganisms, such as other bacteria, fungi, and viruses may be needed to comprehensively understand the etiology of orthopedic infection and their association with clinical features.

## Data availability statement

The raw data supporting the conclusions of this article will be made available by the authors upon request, without undue reservation.

## Ethics statement

The studies involving humans were approved by Qingdao University, Qingdao Huangdao Traditional Chinese Medicine Hospital. The studies were conducted in accordance with the local legislation and institutional requirements. Written informed consent for participation in this study was provided by the participants’ legal guardians/next of kin.

## Author contributions

YNW: Writing – review & editing, Writing – original draft, Validation, Methodology, Investigation, Formal analysis, Data curation. WX: Writing – review & editing, Resources, Methodology, Investigation. YW: Writing – review & editing, Project administration, Methodology, Investigation, Data curation. YC: Writing – review & editing, Resources, Methodology, Investigation. LY: Data curation, Writing – review & editing, Investigation. CL: Writing – review & editing, Investigation, Data curation. DZ: Writing – review & editing, Investigation, Data curation. XG: Resources, Writing – review & editing, Investigation. YDW: Writing – review & editing, Investigation, Data curation. SW: Writing – review & editing, Investigation. JLi: Writing – review & editing, Investigation, Funding acquisition. YL: Writing – review & editing, Methodology, Investigation. JH: Writing – review & editing, Resources, Methodology, Investigation. JLiu: Writing – review & editing, Writing – original draft, Supervision, Methodology, Funding acquisition, Conceptualization.
