# Supplementary material for: Multiplexed bacterial pathogen detection and clinical characteristics of orthopedic infection in hospitalized patients
Source: Front Cell Infect Microbiol. 2024 Jun 13;14:1394352. doi: 10.3389/fcimb.2024.1394352 (PMC11210349; doi:10.3389/fcimb.2024.1394352)
Supplement: Supplementary file 1 [file DataSheet_1.pdf]

Supplementary Material

1 Supplementary Figures and Tables

Table S1. Analytical performance of qPCR panels. Linearity of each assay was tested with a serial 10-fold dilution of positive control materials, and the PCR efficiency was calculated. LOD was determined as the lowest concentration where 100% detection was achieved for 20 repeated testing. CV was calculated to represent repeatability and reproducibility for 20 repeats in one experiment and in five experiments over three days, respectively. Analytical sensitivity and specificity were evaluated on culture isolates, including *Staphylococcus aureus* (64), *Staphylococcus epidermidis* (7), *Staphylococcus haemolyticus* (5), *Staphylococcus simulans* (5), *Staphylococcus hominis* (1), *Staphylococcus caprae* (1), *Acinetobacter baumannii* (3), *Acinetobacter pittii* (2), *Acinetobacter soli* (1), *Aeromonas hydrophila* (2), *Klebsiella aerogenes* (1), *Enterobacter hormaechei* (6), *Enterobacter roggenkampii* (1), *Escherichia coli* (14), *Klebsiella pneumoniae* (10), *Enterococcus faecalis* (2), *Proteus mirabilis* (4), *Proteus penneri* (1), *Proteus vulgaris* (1), *Serratia marcescens* (2), *Streptococcus agalactiae* (2), *Moraxella catarrhalis* (1), *Providencia rettgeri* (1), *Pseudomonas aeruginosa* (7) and *Pseudomonas stutzeri* (2).

	**Pathogen**	**LOD (copies/PCR reaction)**	**PCR efficiency (%)**	**Sensitivity (%)**	**Specificity (%)**	**Repeatability (CV, %)**	**Reproducibility (CV, %)**
Panel 1	*Staphylococcus aureus*	10	99.1	100	100	2.5	1.9
	Acinetobacter baumannii	10	82.7	100	100	2.3	4.0
Panel 2	*Pseudomonas aeruginosa*	10	99.1	100	100	2.3	3.0
	Escherichia coli	10	94.7	93.3	97.9	0.9	1.8
	Haemophilus influenzae	10	86.5	NA	100	1.2	2.6
Panel 3	*Neisseria meningitidis*	10	100.0	NA	100	1.1	1.3
	Klebsiella pneumonia	20	99.5	85.7	100	1.0	1.2
	Klebsiella aerogenes	20	97	NA	99.1	0.9	0.6
Panel 4	*Enterobacter cloacae*	20	93.7	NA	100	1.2	1.0
	Streptococcus pneumoniae	10	80.1	NA	100	1.1	2.1
	Streptococcus pyogenes	10	80.3	NA	100	1.6	3.1
	Streptococcus agalactiae	10	81	100	100	1.4	2.8

NA , no culture isolate was available for the pathogen. Synthetic positive controls were used instead for evaluation.

Table S2. Results of laboratory tests for the patients enrolled in the current study. The test results showing significant difference between the two groups were in bold.

Laboratory test		Total (349)	**Group A (193)**	**Group B (156)**	*P*-value
ABO blood type					**0.015**
	A	92	49	43	
	B	100	55	45	
	O	85	**34**	**51**	
	AB	45	**31**	**14**	
Absolute eosinophil count					0.705
	low	54	31	23	
	normal	290	159	131	
	high	3	1	2	
Absolute lymphocyte count					0.159
	low	19	14	5	
	normal	327	176	151	
	high	1	1	0	
Absolute neutrophil count					0.832
	low	8	5	3	
	normal	251	136	115	
	high	88	50	38	
Absolute value of basophils					1
	low	0	0	0	
	normal	345	189	156	
	high	1	1	0	
Absolute value of immature granulocytes					**0.018**
	low	0	0	0	
	normal	303	165	138	
	high	9	**9**	**0**	
Absolute value of monocytes					0.107
	low	1	0	1	
	normal	318	171	147	
	high	28	20	8	
Adenylic deaminase					0.265
	low	3	2	1	
	normal	322	176	146	
	high	13	10	3	
Alanine aminotransferase					**0.021**
	low	0	0	0	
	normal	292	153	139	
	high	50	**35**	**15**	
Albumin					**0.003**
	low	65	**48**	**17**	
	normal	272	140	132	
	high	1	0	1	
Alkaline phosphatase					0.178
	low	0	0	0	

	normal	312	168	144	
	high	30	20	10	
Amylase					0.922
	low	0	0	0	
	normal	329	181	148	
	high	1	0	1	
Blood platelet count					**0.009**
	low	5	4	1	
	normal	293	151	142	
	high	49	**36**	**13**	
Calcium					0.43
	low	88	52	36	
	normal	253	135	118	
	high	1	1	0	
Carbon dioxide binding rate					0.307
	low	61	29	32	
	normal	247	139	108	
	high	30	19	11	
Chlorine					0.316
	low	2	2	0	
	normal	324	177	147	
	high	12	8	4	
C-reactive protein					0.853
	low	0	0	0	
	normal	62	42	20	
	high	98	65	33	
Creatinine					0.383
	low	162	91	71	
	normal	178	95	83	
	high	2	2	0	
Creatine kinase					**<0.001**
	low	25	**21**	**4**	
	normal	189	111	78	
	high	115	**49**	**66**	
D-Dimer					0.085
	low	0	0	0	
	normal	198	112	86	
	high	102	47	55	
Direct bilirubin					0.496
	low	0	0	0	
	normal	302	164	138	
	high	40	24	16	
Erythrocyte sedimentation rate					0.726
	low	0	0	0	
	normal	40	27	13	
	high	85	60	25	
Fucosidase					0.739
	low	0	0	0	
	normal	315	170	145	
	high	19	11	8	

Globulin					0.411
	low	24	11	13	
	normal	313	176	137	
	high	1	1	0	
α1globulin					**0.002**
	low	1	0	1	
	normal	236	116	120	
	high	81	**57**	**24**	
α2globulin					**0.009**
	low	13	4	9	
	normal	258	135	123	
	high	47	**34**	**13**	
β-globulin					0.113
	low	7	6	1	
	normal	290	153	137	
	high	21	14	7	
γ-globulin					0.293
	low	3	2	1	
	normal	223	115	108	
	high	92	56	36	
Glucose					0.197
	low	3	3	0	
	normal	247	138	109	
	high	91	46	45	
Glutamic oxalacetic transaminase					0.155
	low	0	0	0	
	normal	309	166	143	
	high	33	22	11	
Glutamic oxalacetic transaminase/ glutamic-pyruvic transaminase					0.524
	low	90	54	36	
	normal	245	130	115	
	high	7	4	3	
γ-glutamyl transpeptidase					**0.046**
	low	14	8	6	
	normal	282	147	135	
	high	46	**33**	**13**	
Hemoglobinometry					0.112
	low	30	20	10	
	normal	299	158	141	
	high	18	13	5	
Hematocrit value					0.655
	low	46	26	20	
	normal	294	160	134	
	high	7	5	2	
High density lipoprotein cholesterol					**0.035**
	low	110	**70**	**40**	
	normal	186	98	88	
	high	45	19	26	
Human serum amyloid					-

low	0	0	0	
normal	1	1	0	
high	4	4	0	
α-hydroxybutyrate Dehydrogenase				0.256
low	6	4	2	
normal	306	164	142	
high	18	13	5	
Indirect bilirubin				0.089
low	0	0	0	
normal	286	163	123	
high	56	25	31	
Kalium				0.732
low	28	16	12	
normal	307	170	137	
high	3	1	2	
Lactic dehydrogenase				0.504
low	4	3	1	
normal	268	143	125	
high	59	35	24	
Large platelet count				0.292
low	32	22	10	
normal	273	148	125	
high	7	4	3	
Lipoprotein				0.428
low	0	0	0	
normal	255	143	112	
high	86	44	42	
Low density lipoprotein cholesterin				0.068
low	0	0	0	
normal	249	144	105	
high	92	43	49	
Magnesium				0.504
low	0	0	0	
normal	333	183	150	
high	2	2	0	
Mean corpuscular hemoglobin				0.618
low	13	8	5	
normal	175	92	83	
high	159	91	68	
Mean corpuscular protein concentration				0.872
low	14	8	6	
normal	333	183	150	
high	0	0	0	
Mean corpuscular volume				0.74
low	12	7	5	
normal	260	140	120	
high	75	44	31	
Mean platelet volume				-
low	0	0	0	
normal	347	191	156	

	high	0	0	0	
Natrium					0.332
	low	18	12	6	
	normal	319	175	144	
	high	1	0	1	
Nucleated red blood cells					0.505
	low	0	0	0	
	normal	310	172	138	
	high	2	2	0	
Percentage of basophils					0.86
	low	0	0	0	
	normal	344	190	154	
	high	3	1	2	
Percentage of eosinophils					0.33
	low	104	52	52	
	normal	233	132	101	
	high	10	7	3	
Percentage of immature granulocytes					0.198
	low	0	0	0	
	normal	308	170	138	
	high	4	4	0	
Percentage of lymphocytes					0.725
	low	152	80	72	
	normal	176	100	76	
	high	19	11	8	
Percentage of monocytes					0.755
	low	10	6	4	
	normal	274	148	126	
	high	63	37	26	
Percentage of neutrophils					0.303
	low	12	8	4	
	normal	241	137	104	
	high	94	46	48	
Percentage of nucleated red blood cells					0.505
	low	0	0	0	
	normal	310	172	138	
	high	2	2	0	
Phosphorus					0.724
	low	12	6	6	
	normal	311	170	141	
	high	19	12	7	
Platelet distribution width					1
	low	0	0	0	
	normal	343	189	154	
	high	4	2	2	
Platelet large cell ratio					0.22
	low	13	10	3	
	normal	329	179	150	
	high	5	2	3	

Prealbumin					**<0.001**
	low	125	**87**	**38**	
	normal	210	100	110	
	high	3	1	2	
Procalcitonin					**0.03**
	low	0	0	0	
	normal	29	25	4	
	high	46	**29**	**17**	
RBC coefficient of variation					0.132
	low	0	0	0	
	high	336	182	154	
	normal	11	9	2	
Red blood cell count					0.4
	low	19	13	6	
	normal	321	175	146	
	high	7	3	4	
Ratio of albumin to globulin					0.054
	low	41	**30**	**11**	**0.016**
	normal	291	155	136	
	high	6	3	3	
RBC standard deviation					0.499
	low	1	0	1	
	normal	343	189	154	
	high	3	2	1	
Rh blood group					0.592
	positive	314	166	148	
	negative	6	2	4	
Sialic acid					**0.002**
	low	5	3	2	
	normal	229	112	117	
	high	103	**72**	**31**	
Thrombocytocrit					0.132
	low	6	5	1	
	normal	300	159	141	
	high	40	26	14	
Total cholesterol					**0.026**
	low	0	0	0	
	normal	223	132	91	
	high	118	**55**	**63**	
Total bile acid					1
	low	0	0	0	
	normal	330	184	146	
	high	8	4	4	
Total bilirubin					0.261
	low	0	0	0	
	normal	284	160	124	
	high	58	28	30	
Total protein					0.079
	low	64	**43**	**21**	**0.035**
	normal	270	142	128	

	high	4	3	1	
Triglyceride					0.871
	low	4	2	2	
	normal	271	147	124	
	high	66	38	28	
Urea nitrogen					0.27
	low	12	7	5	
	normal	301	161	140	
	high	29	20	9	
Uric Acid					0.619
	low	32	20	12	
	normal	257	138	119	
	high	53	30	23	
White blood cell count					0.318
	low	9	7	2	
	normal	274	147	127	
	high	64	37	27	

Table S3 Comparison of demographic and clinical features between patients with pathogens identified and those without. The results showing significant difference between the two groups were in bold. For pathogen detection by qPCR, Cq cutoff of 35 was used to determine the patients with one or more bacterial pathogen except that Ct 28.7 was used for *P. aeruginosa* and 34.4 for *K. pneumoniae*, While Cq cutoff of 38.5 was used to ensure no pathogen detected.

	Patients with 12 bacterial pathogens detected by either culture or qPCR (n = 81)	Patients with no pathogen detected by both culture and qPCR (n = 95)	Total (n = 176)	P-value	OR (95% CI)
**Gender**			176	**0.027**	2.2 (1.1, 4.3)
Male	65	62	127		
Female	16	33	49		
**Age (yr)**	52.9 ± 15.9	50.5 ± 17.3		0.340	
**Length of stay (day)**	15.0 (10.0, 31.0)	13.0 (7.0, 30.0)	15.0 (8.0, 30.8)	0.203	
**Underlying disease**	37	45	82	0.823	
Previous orthopedic surgery	13	6	19	**0.038**	**2.8 (1.0, 7.8)**
Hypertension	12	18	30	0.467	
Diabetes	8	13	21	0.437	
**Clinical manifestations**					
Redness	34	30	64	0.153	1.6 (0.8, 2.9)
Purulent exudate	13	7	20	0.071	2.4 (0.9, 6.4)
Swelling	53	61	114	0.866	1.1 (0.6, 2.0)
Bleeding	14	34	48	**0.006**	0.4 (0.2, 0.8)
Pain	73	88	161	0.553	0.7 (0.3, 2.1)
Malformed	13	11	24	0.389	1.5 (0.6, 3.5)
Restrictied activity	51	66	117	0.362	0.7 (0.4, 1.4)
Tissues exposed	1	10	11	**0.011**	0.1 (0.0, 0.8)
Contaminated wound	7	16	23	0.108	0.5 (0.2, 1.2)
Bone exposure	0	5	5	0.101	-
Diabrosis	21	20	41	0.446	1.3 (0.7, 2.6)
Seepage	35	27	62	**0.041**	1.9 (1.0, 3.6)
High skin temperature	27	19	46	**0.045**	2.0 (1.0, 4.0)
Subcutaneous hemorrhage	7	8	15	0.958	1.0 (0.4, 3.0)
Effusion	5	4	9	0.556	1.5 (0.4, 5.8)

Open wound	23	40	63	0.059	0.5 (0.3, 1.0)
Necrosis	6	3	9	0.351	2.5 (0.6, 10.1)
Implant exposure	1	0	1	0.46	-
Unhealed incision	4	3	7	0.829	1.6 (0.3, 7.3)
Poor circulation	2	13	15	**0.008**	0.2 (0.0, 0.7)
Absolute lymphocyte count			174	0.624	
low	7	7	14		
normal	73	86	159		
high	0	1	1		
Absolute neutrophil count			174	0.351	
low	1	4	5		
normal	59	62	121		
high	20	28	48		
Absolute value of monocytes			174	0.524	
low	0	0	0		
normal	73	83	156		
high	7	11	18		
Absolute value of immature granulocytes			160	0.827	
low	0	0	0		
normal	73	79	152		
high	3	5	8		
Adenylic deaminase			171	0.283	
low	0	1	1		
normal	78	84	162		
high	2	6	8		
Alanine aminotransferase			171	0.116	
low	0	0	0		
normal	60	77	137		
high	20	14	34		
Albumin			171	0.631	
low	21	21	42		
normal	50	70	129		
high	0	0	0		
Alkaline phosphatase			171	0.317	

low	0	0	0	
normal	74	80	154	
high	6	11	17	
Blood platelet count			174	0.399
low	2	1	3	
normal	60	78	138	
high	18	15	33	
C-reactive protein			99	0.896
low	0	0	0	
normal	19	18	37	
high	31	31	62	
Creatine kinase			166	0.279
low	9	10	19	
normal	51	48	99	
high	18	30	48	
D-Dimer			144	0.571
low	0	0	0	
normal	46	54	100	
high	18	26	44	
Erythrocyte sedimentation rate			79	0.975
low	0	0	0	
normal	11	12	23	
high	27	29	56	
Fucosidase			166	**0.048**
low	0	0	0	
normal	76	79	155	
high	2	9	11	
Globulin			171	0.449
low	3	6	9	
normal	77	84	161	
high	0	1	1	
Glutamic oxalacetic transaminase			171	0.746
low	0	0	0	
normal	69	80	149	

	high	11	11	22		
Glutamic oxalacetic transaminase/ glutamic-pyruvic transaminase				171	0.663	
	low	24	28	52		
	normal	55	60	115		
	high	1	3	4		
Hemoglobinometry				174	0.062	
	low	13	5	18	**0.019**	3.4 (1.2, 10.2)
	normal	62	82	144		
	high	5	7	12		
High density lipoprotein cholesterin				170	0.357	
	low	33	30	63		
	normal	41	51	92		
	high	5	10	15		
Lactic dehydrogenase				166	**0.033**	
	low	1	2	3		
	normal	68	62	130		
	high	9	24	33	**0.01**	0.3 (0.1, 0.8)
Low density lipoprotein cholesterin				170	0.437	
	low	0	0	0		
	normal	63	68	131		
	high	16	23	39		
Percentage of lymphocytes				174	0.663	
	low	33	42	75		
	normal	43	45	88		
	high	4	7	11		
Percentage of monocytes				174	0.512	
	low	3	2	5		
	normal	59	76	135		
	high	18	16	34		
Percentage of neutrophils				174	0.46	
	low	2	6	8		
	normal	59	65	124		
	high	19	23	42		
Prealbumin				171	0.083	

	low	44	37	81		
	normal	35	54	89		
	high	1	0	1		
Procalcitonin				50	0.615	
	low	0	0	0		
	normal	11	11	22		
	high	12	16	28		
Ratio of albumin to globulin				171	0.448	
	low	16	12	28		
	normal	63	77	140		
	high	1	2	3		
Red blood cell count				174	**0.026**	
	low	10	2	12	**0.007**	6.6 (1.4, 31.1)
	normal	69	91	160		
	high	1	1	2		
α1 globulin				158	0.338	
	low	0	0	0		
	normal	47	58	105		
	high	28	25	53		
α2 globulin				158	0.867	
	low	2	2	4		
	normal	57	66	123		
	high	16	15	31		
β globulin				158	0.805	
	low	3	2	5		
	normal	66	73	139		
	high	6	8	14		
γ globulin				158	0.972	
	low	1	1	2		
	normal	50	54	104		
	high	24	28	52		
Sialic acid				170	**0.02**	
	low	1	2	3		
	normal	38	62	100		

	high	40	27	67	**0.006**	2.4 (1.3, 4.6)
Total bilirubin				171	0.791	
	low	0	0	0		
	normal	68	76	144		
	high	12	15	27		
Total cholesterol				170	0.451	
	low	0	0	0		
	normal	58	62	120		
	high	21	29	50		
Total protein				171	0.82	
	low	19	19	38		
	normal	60	70	130		
	high	1	2	3		
γ-glutamyl transpeptidase				171	0.613	
	low	2	5	7		
	normal	63	69	132		
	high	15	17	32		
White blood cell count				174	0.284	
	low	2	5	7		
	normal	65	67	132		
	high	13	22	35		
